# Mapping Manual Laboratory Tasks to Robot Movements in Digital Pathology Workflow

**DOI:** 10.3390/s25226830

**Published:** 2025-11-08

**Authors:** Marianna Dimitrova Kucarov, Mátyás Takács, Bence Géza Czakó, Béla Molnár, Miklos Kozlovszky

**Affiliations:** 1 Doctoral School of Applied Informatics and Applied Mathematics, Obuda University, 1034 Budapest, Hungary; 2 BioTech Research Center, Obuda University, 1034 Budapest, Hungary; kozlovszky.miklos@uni-obuda.hu; 3 3DHistech Ltd., 1141 Budapest, Hungary; matyas.takacs@3dhistech.com (M.T.); molnar.bela@semmelweis.hu (B.M.); 4 Antal Bejczy Center for Intelligent Robotics (IROB), Obuda University, 1034 Budapest, Hungary; 5 Turbine Ltd., 1083 Budapest, Hungary; bence.czako@turbine.ai; 6 Department of Internal Medicine and Oncology, Faculty of Medicine, Semmelweis University, 1085 Budapest, Hungary; 7 John von Neumann Faculty of Informatics, Obuda University, 1034 Budapest, Hungary; 8 Medical Device Research Group, LPDS, Institute for Computer Science and Control (SZTAKI), Hungarian Research Network (HUN-REN), 1111 Budapest, Hungary

**Keywords:** applied robotics, tissue staining, coverslipping and scanning, integrating pathology equipment, mapping manual laboratory tasks to robot movements, autonomous pathology workflow, slide and magazine manipulation

## Abstract

This study evaluated and integrated automatic pathology equipment and a collaborative robot to create a fully autonomous workflow. We selected the Gemini AS Automated Slide Stainer, ClearVue Coverslipper, and Pannoramic 1000 digital slide scanner, controlled by a UR5e robotic arm. To perform essential clinical laboratory tasks, we determined that the robotic arm, in combination with a custom manipulator, requires 9 degrees of freedom—5 from the robot and 4 from the manufactured manipulator. The patented manipulator is equipped with a camera, LED lighting, and three specialized grippers for object detection and precise handling of equipment doors, magazines, and slides. It is designed to mount onto a standardized robot flange interface (ISO 9409-1-50-4-M6), making it mechanically compatible with various robot arms. A minimum of 24 distinct laboratory tasks were defined for the training of the robotic arm. This autonomous workflow mitigates labor shortages and accelerates diagnostic processes by offloading repetitive tasks, thereby improving efficiency in pathology laboratories.

## 1. Introduction

Digital pathology involves the management and interpretation of pathological information generated from digitized glass slides. This transformation from traditional microscopy to digital imaging offers numerous advantages, including enhanced collaboration among pathologists, improved accessibility to cases, and the potential for integration with artificial intelligence (AI) for diagnostic support and quantitative analysis. The digitization of pathology workflows forms a crucial foundation for advancing diagnostic precision and efficiency within the field.

The digital pathology workflow commences with tissue preparation, a labor-intensive process involving fixation, grossing, tissue processing, and paraffin embedding to preserve tissue morphology. This is followed by sectioning, where the embedded tissue blocks are cut into very thin sections using a microtome. These sections are then placed on glass slides and undergo staining, most commonly with Hematoxylin and Eosin (H&E), but also potentially with special stains or immunohistochemistry (IHC) to highlight specific cellular components or markers [1,2]. After staining, the slides are coverslipped for permanent preservation and undergo initial quality control. The final step in this initial sequence is whole slide imaging (WSI), where a high-resolution scanner converts the prepared glass slides into digital images, effectively digitizing the tissue for subsequent digital review [3,4].

The rationale for automating digital pathology workflows is rooted in addressing the inefficiencies and variability inherent in manual processes. Traditional pathology laboratories frequently encounter labor-intensive tasks, including slide handling, scanning, and data management. Such manual steps are prone to human error, can introduce inconsistencies, and ultimately limit throughput, especially in high-volume environments. Automation mitigates these challenges by introducing robotic precision and consistency, thereby reducing turnaround times, minimizing errors, and enabling laboratories to process a greater volume of cases without a commensurate increase in human resources. The integration of robotic systems has demonstrably enhanced reproducibility and standardization across various analytical processes, paving the way for similar advancements in digital pathology [5,6]. Consequently, as the field of pathology has transitioned toward digital pathology over the past four decades, leading international companies such as Leica Biosystems, Koninklijke Philips N.V., Roche, PathAI, Aiforia Technologies, and 3DHistech [7] have begun to incorporate robotization into their diagnostic and treatment workflows to address identified bottlenecks [8].

The primary objective of this paper is to address the challenges associated with the final step in the automation workflow: the transfer of slides between the coverslipper and the scanner. Currently, no integrated commercial solution provides a direct link between these two independent instruments, largely due to fundamental design variations among different manufacturers. An ideal system would utilize a single, compatible magazine to hold and transfer slides between the coverslipper and the scanner. However, the mechanical layouts of the magazines, as well as the slide ejection and insertion mechanisms, differ significantly across manufacturers, thus necessitating the manual transfer of slides between distinct magazine types. This highlights the need for a highly flexible automation solution, which can be achieved using a robotic arm equipped with a suitable gripper mechanism. In this paper, we provide a detailed breakdown of the individual steps required for the robotic transition of slides between the coverslipper and scanner. We also specify the number of degrees of freedom (DOF) needed for each step. Our guidelines are based on the experiences gained from developing and implementing our own in-house solution.

Our primary contribution is the provision of guidelines designed to optimize resource utilization and achieve cost efficiencies in pathological laboratory automation. The findings presented here can inform the design of robotic systems that are tailored, efficient, and economically feasible. By advocating for a standardized method of workflow automation, this research supports a broader initiative to standardize digital pathology processes across various institutions and commercial entities. Such standardization is crucial for enabling interoperability, facilitating data exchange, and promoting the wider integration of artificial intelligence-powered diagnostic tools in pathology.

This study operates under certain limitations. The proposed guidelines are specifically tailored for small to mid-sized laboratories, where flexibility in workflow device arrangement is often a key consideration. The recommendations assume a flexible arrangement rather than a rigidly fixed setup, allowing for adaptability to various laboratory layouts and equipment types. Furthermore, the description is intentionally component-agnostic, meaning it does not prescribe specific brands or models of equipment. While this offers flexibility in terms of procurement and allows laboratories to acquire components based on available funds, it may result in a slightly slower pipeline compared to a highly specialized, integrated system. This flexibility, however, ensures broader applicability and facilitates phased implementation based on budgetary constraints.

Beyond this study, a substantial body of literature addresses pathological laboratory automation, with a notable focus also on streamlining diagnostic workflow. Existing research offers recommendations for standardizing analytical and bioanalytical laboratories, highlighting how these standardized methods can enhance throughput and improve the quality of results [9,10,11]. Specifically, several papers provide overviews of pathologist workflows [12,13] and demonstrate concrete implementations of automated systems [14]. Furthermore, research has been conducted on specialized aspects, such as the robotic handling of pathological slides [15].

The core message of this paper is that a robotic arm possessing nine degrees of freedom is sufficient for automating the slide handling between the coverslipper and the scanner. The determination of the absolute lowest number of degrees of freedom required for automation is paramount due to its direct correlation with the cost and the complexity of the robotic system. Robots with more degrees of freedom are inherently more expensive and may introduce unnecessary mechanical intricacy. Therefore, identifying the irreducible minimum DOF for each component is crucial for achieving an economically viable and functionally efficient automation solution. This consideration is particularly significant for the end-effector or tool mounted on the robotic arm, as specific movements and orientations are mandated by the diverse appliances and instrumentation within the digital pathology workflow (e.g., slide scanners, coverslippers, and automated staining platforms). Optimizing DOF ensures that the robotic system can perform all necessary actions—such as precise gripping, linear translation, and rotational manipulation—without over-engineering, thereby minimizing capital expenditure and operational complexity.

Following this introduction, Section 2 provides an overview of commercially available tissue stainers, coverslippers, scanner types, and robotic arms. Section 3 details the subtasks involved in magazine and slide transportation and determines the number and types of DOF required for each. Finally, in Section 4, we discuss the results and limitations in detail while also proposing future directions for improved efficiency.

## 2. Materials and Methods

This section provides an overview of prominent, commercially available devices that are essential for automating pathology workflows. The selection is restricted to instruments readily adaptable for this purpose. While a comprehensive effort was made to compile these lists, we acknowledge that other suitable appliances for automation may exist. A dedicated comparison of tissue stainers is included to demonstrate that integrated automation solutions sometimes exist between tissue stainers and coverslippers, thereby obviating the need for a robotic arm for this specific task. Following this comparative analysis, the subtasks associated with automating each device are individually defined, along with the specific types of robotic arm movements required for each subtask. We have intentionally excluded the financial costs associated with each device from this comparison. This decision is based on the understanding that pricing can vary significantly due to individual business agreements, regional market fluctuations, and applicable tax regulations. Consequently, the inclusion of cost data could introduce variability that might misrepresent the economic considerations for different laboratories.

### 2.1. Staining Tissue Samples

Tissue staining represents a foundational procedure in histopathology, designed to augment the contrast of cellular and structural elements for subsequent microscopic examination. The integration of automated tissue stainers in laboratory settings is extensive, serving to standardize and expedite this process. A detailed comparison of prominent international models, differentiated by manufacturer, is presented in Table 1. This table delineates essential characteristics such as the model type and vendor, maximum number of magazines, slide capacity of magazine, maximum throughput, supported staining protocols, special features, and system compatibility. The provided data is instrumental for assessing each manufacturer’s design principles and selecting a system that aligns with the specific requirements of a given laboratory.

For pathology laboratories requiring high throughput combined with flexible protocol execution, the Sakura **Tissue-Tek Prisma Plus Automated Slide Stainer** (Tokyo, Japan) offers a compelling solution. This instrument delivers industry-leading slide processing speed of up to 530 slides per hour, supported by an intelligent scheduler that minimizes idle time while maintaining consistent staining quality. It supports continuous loading and can run up to 11 staining protocols simultaneously. Operational advantages include built-in barcode reading to scan and log stain kits and reagent components, track expiration dates and lot numbers, and support CAP accreditation compliance. The platform includes Tissue-Tek iSupport for remote diagnostics and faster issue resolution, thus enhancing uptime and minimizing interruptions. Capacity-wise, Prisma Plus accepts baskets of 10 or 20 slides per run, with continuous loading across up to three start stations, enabling a typical run size of 60 slides per protocol execution. With up to 51 reagent reservoirs and optional heating and drying stations, the system accommodates both routine Hematoxylin–Eosin (H&E) and special staining workflows with minimal intervention. Integration with Sakura’s Tissue-Tek Film Coverslipper or Glas g2 Coverslipper creates a fully automated end-to-end workflow from slide baking through drying and coverslipping [16].

The second stainer in our comparison is the Leica **HistoCore SPECTRA ST Stainer** (Nussloch, Germany), which also represents cutting-edge staining technology. This stainer features a substantial capacity, accommodating 12 racks, each capable of holding 30 slides for routine staining or 5 slides for special stains. It can achieve a throughput of up to 360 slides per hour, facilitated by an integrated dual translational robotic arm. A key operational advantage is its ability to store and simultaneously execute both H&E and special staining procedures, thereby providing considerable flexibility [17]. The platform has a built-in barcode system that permits the precise tracking of different slides. The device also incorporates an integrated oven, which streamlines the workflow by eliminating the requirement for an external heating unit. The stainer is designed for straightforward integration with the Leica HistoCore SPECTRA CV Coverslipper, and it is also available as a standalone HistoCore SPECTRA workstation. This workstation further automates the transfer between the staining and coverslipping stages, consequently decreasing the turnaround time between these subtasks [18].

Our third compared device is the Epredia **Gemini™AS** (Kalamazoo, Michigan, USA), characterized by its compact, dual-level configuration, which is capable of managing 10–15 racks, each with a capacity of 20 slides, thereby achieving a throughput ranging from 200 to 300 slides per hour. A distinguishing feature of the Gemini AS is its STAT function, which permits the prioritization of samples for expedited staining. The model is engineered for seamless integration with its respective coverslipper, the Epredia ClearVue, to establish a comprehensive, automated workflow [19]. This device does not include an integrated heater, and the connection to the coverslipper necessitates external automation, similar to the Tissue-Tek Prisma Plus Automated Slide Stainer.

Finally, a distinct approach to automation is offered by Guangdong Jinquan’s **JQ-DS200** (Shenzhen, Guangdong, China) model. This system is designed as an all-in-one unit, integrating both staining and coverslipping functionalities. This design eliminates the necessity for separate coverslippers, which is particularly beneficial for laboratories with spatial constraints. The JQ-DS200 provides a throughput of 200 slides per hour. It supports H&E and special stains, and a notable feature is its capacity to operate without xylene or alcohol, thereby fostering a more environmentally friendly and safer laboratory environment. The system also incorporates a built-in barcode system for tracking the quantity of slides within the device, and includes a priority staining feature, analogous to the STAT function found in the Gemini AS [20,21].

All the aforementioned stainers are capable of performing the required protocols, which include H&E [22] and Giemsa [23] for cytoplasm and nuclei visualization, as well as $4^{\prime }$,6-diamidino-2-phenylindole (DAPI) [24] for nuclei contrasting in fluorescence applications. Therefore, the final decision was determined by laboratory requirements (maximum throughput, supported staining protocols), system compatibility, and cost (customs tariffs) in Hungary (excluded from Table 1). Based on this comparative analysis, the Gemini AS Automated Slide Stainer was ultimately selected for our case study.

### 2.2. Coverslipping Tissue Samples

Coverslipping constitutes a critical step subsequent to the staining process, involving the sealing of stained tissue samples, typically with a thin glass coverslip. This procedure fulfills two primary objectives: to preserve the sample within a contamination-free and air-free environment for long-term archival and subsequent reuse, and to establish a consistent optical path conducive to clear microscopic examination. Given that coverslipping serves as a complementary service to tissue staining, the predominant entities in automated stainer manufacturing frequently also lead in coverslipping technology. As elaborated in Table 2, a comparative analysis of these prominent international models reveals a diverse array of key features. The selection of a coverslipper is contingent upon factors such as operational speed, the type of coverslip employed, and the potential for integration with pre-existing staining systems.

The Sakura **Tissue-Tek Film Automated Coverslipper** (Tokyo, Japan) is a high-speed solution that forgoes traditional glass coverslips in favor of a special coverslipping film. This method offers an impressive throughput of up to 1080 slides per hour and eliminates the need for liquid mounting medium, resulting in a dry, bubble-free slide in approximately four seconds. Its system is designed for seamless integration with the Tissue-Tek Prisma Plus Automated Slide Stainer, creating an efficient and fully automated workflow [25].

By contrast, the Sakura **Tissue-Tek Glas g2 Automated Glass Coverslipper** (Tokyo, Japan) provides a more traditional glass-based solution with a maximum capacity of 400 slides per hour. It features a precise dispensing method for mounting medium to minimize air bubbles, catering to laboratories that prefer the conventional glass coverslip and liquid mounting medium approach [26].

For laboratories utilizing Leica stainers, the Leica **HistoCore SPECTRA CV** (Nussloch, Germany) coverslipper provides integrated solutions. This coverslipper distinguishes itself as the sole system in its category featuring dual independent glass-coverslip lines, enabling a coverslipping throughput of up to 570 dried slides per hour. An integrated hot-air oven facilitates the production of touch-dry slides in approximately five minutes, which allows for immediate handling and reduces turnaround time. The robust, continuous workflow is supported by a nine-rack output drawer with a 270-slide capacity, RFID tracking, and an automated broken-glass detector, all contributing to enhanced safety and quality assurance. However, it should be noted that as of 2025, this coverslipper is not available for purchase as a standalone item; it is only offered as part of the fully integrated HistoCore SPECTRA Workstation system, which includes the HistoCore SPECTRA ST Stainer [18].

The Epredia **ClearVue Coverslipper** (Kalamazoo, Michigan, USA) is designed with intelligent control software and a throughput of up to 250 slides per hour. Gemini coverslip 11 slide baskets, accepts slides directly from the Epredia Gemini AS, Varistain 24-4, Sakura DRS2000, and Leica autostainer. A notable feature is its advanced optical recognition, which automatically positions slides and dispenses the correct amount of mounting medium for both histology and cytology samples without user intervention [27].

Finally, a unique all-in-one solution is offered by the Guangdong Jinquan **JQ-DS200** (Shenzhen, Guangdong, China), as previously noted in Section 2.1. These systems integrate both staining and coverslipping functionalities into a single unit, which is particularly advantageous for laboratories with restricted bench space. The throughput of the coverslipping component aligns with that of the staining component due to their interconnected design, processing 200 glass slides per hour [20].

In summary, the selection of an automated coverslipper is a strategic decision that must balance throughput requirements, preferred coverslipping methods (film vs. glass), and compatibility with existing equipment to optimize laboratory efficiency and slide quality. As the Epredia Gemini AS Automated Slide Stainer was chosen for the staining process in our case study, the compatible Epredia ClearVue Coverslipper was selected to complete the workflow.

### 2.3. Scanning Tissue Samples

The field of digital pathology fundamentally relies on a diverse array of imaging technologies to transform physical tissue samples into high-resolution digital images. Table 3 provides a general overview by categorizing leading scanners currently available in the market based on their primary function and the underlying imaging principles they employ. We have included several illustrative examples for each class; however, it is important to note that numerous other models are also available on the market, rendering our list demonstrative rather than exhaustive.

The most prevalent and broadly adopted instruments are whole slide scanners. These devices are engineered for high-throughput acquisition of entire glass slides, generating comprehensive digital images suitable for computer-based viewing and analysis. Within this classification, brightfield scanners, exemplified by the Leica Aperio GT450DX [28], 3DHISTECH Pannoramic 1000 [29], Phillips Pathology Scanner SG300 [30], and Hamamatsu NanoZoomer S360 [31], represent the established industry standard for conventional histology, employing visible light to capture images of stained tissue. For more specialized applications, fluorescence scanners, such as the Visialab EPIQO [32], are utilized to visualize specific biomarkers with fluorescent dyes. Multimodal, or “hybrid,” scanners, including the Roche Ventana DP 600 [33], ZEISS Axioscan [34], and Akoya Biosciences PhenoImager HT [35], integrate both brightfield and fluorescence capabilities within a single system, thereby providing augmented versatility.

Beyond standard whole slide imaging, specialized systems address specific research and clinical requirements. Advanced Research Imaging platforms, such as confocal and multiphoton microscopes (e.g., Nikon AX [36], ZEISS LSM 990 NLO [37]), are generally not employed for whole slide scanning. However, they are indispensable for producing high-resolution, three-dimensional images of cellular structures. Tissue Microarray (TMA) analysis represents a distinct application focused on the scanning and analysis of numerous small tissue samples arrayed in a single block. Platforms like the 3DHISTECH TMA Module [38] are specifically designed for this high-throughput technique.

For specialized applications, other distinct technologies have emerged. Optical Coherence Tomography (OCT) systems, such as the Thorlabs Vega Series SS-OCT [39] and Heidelberg OCT Spectralis [40], employ light to acquire cross-sectional images of biological tissue in situ. This provides a non-invasive, real-time visualization of tissue architecture. In a separate domain, intraoperative and handheld devices, including the Grundium Ocus 40 microscope slide scanner [41] and OptraSCAN OS-FS Frozen Section Scanner [42], offer portability and rapid scanning functionalities. These capabilities are particularly beneficial for point-of-care diagnostics and utilization within surgical environments.

The increasing integration of artificial intelligence within pathology has led to the emergence of a new category of AI-based software platforms that function as decision support systems for already scanned slides. Companies such as Paige AI [43], PathAI [44], and Aiforia [45] offer advanced algorithms for image analysis, feature detection, and diagnostic assistance. These platforms do not operate as scanners themselves; rather, they are crucial for realizing the full potential of data produced by various scanning devices, thereby improving diagnostic accuracy and efficiency.

In conclusion, the landscape of digital pathology is characterized by a wide variety of hardware and software, each optimized for specific tasks. The choice of a system depends on the required throughput, imaging modality, and the specific diagnostic or research questions being addressed.

In the predominant commercial applications within digital pathology, brightfield scanners are extensively employed. These instruments are characterized by their superior throughput capabilities compared to other scanner categories, making them particularly well-suited for integration into routine pathologist workflows. To provide a focused analysis on this widely adopted technology, a dedicated comparison of leading brightfield scanners is presented in Table 4. This specialized table offers detailed insights into their performance characteristics and features relevant to their widespread use in diagnostic settings.

The **Pannoramic 1000** (Budapest, Hungary), manufactured by 3DHISTECH, is engineered for high-throughput operations within research laboratories, distinguished by its exceptional speed and substantial capacity. It achieves a scan of a 15 mm × 15 mm area at 40× magnification in a mere 25 s, positioning it as one of the fastest systems currently available. With an impressive 1000-slide capacity, this scanner is optimally suited for large-scale projects, accommodating both standard and double-width (mega) slides. The system delivers an outstanding resolution of 0.12 µm/pixel at 40× magnification, enabled by its 0.95 numeric aperture, and incorporates an anti-vibration granite base to ensure image stability and quality. Advanced features include an optional 40× water immersion objective for enhanced resolution, and Z-stack imaging for multi-layer analysis [46].

Hamamatsu’s **NanoZoomer S360** (Hamamatsu, Japan) represents a versatile scanning solution, primarily employed in clinical environments. It is capable of scanning a 15 mm × 15 mm area at 40× magnification in approximately 30 s and offers a substantial 360-slide capacity. The NanoZoomer S360 provides resolutions of 0.46 µm/pixel at 20× magnification and 0.23 µm/pixel at 40× magnification. It supports both 20× and 40× magnification modes, with its Z-stack scanning functionality proving particularly valuable for the analysis of thick or complex tissues, thereby furnishing a three-dimensional perspective. The system seamlessly integrates into digital workflows through automatic calibration and support for 1D and 2D barcodes. An optional fluorescence imaging module is also available, enabling the superposition of brightfield and fluorescent images [31].

The **TissueScope iQ** (St. Jacobs, Ontario, Canada) from Huron is a scanner chiefly oriented towards research applications, exhibiting a scan speed of 30 s for a 15 mm × 15 mm area at 40× magnification. It provides a resolution of 0.2 µm/pixel at 40× magnification. This scanner is notable for its operational flexibility, featuring a 400-slide capacity that can accommodate a combination of standard and double-wide slides via its five slide cartridges. It utilizes a non-proprietary 24-bit RGB Pyramidal BigTIFF file format, ensuring broad compatibility, and also supports DICOM. Key functionalities encompass one-click automatic and manual scan modes, Z-stack scanning, and image-based barcode recognition [47].

The Leica **Aperio GT450DX** (Nussloch, Germany) is a high-throughput digital pathology scanner engineered for clinical deployment. It achieves a rapid scanning speed of 32 s for a 15 mm × 15 mm area at 40× magnification and offers a capacity of 450 slides. The system provides a resolution of 0.26 µm/pixel at 40× magnification. This scanner is distinguished by its continuous loading capability, which permits the addition of new slide racks during ongoing scanning operations, and an automated image quality assessment. Furthermore, it supports DICOM-compatible files, Z-stacking, and manual scan modes, and incorporates the Aperio iQC software, which leverages artificial intelligence to detect digital and histological artifacts [48].

Philips’ **Pathology Scanner SG300** (Amsterdam, Netherlands) constitutes a robust solution for both clinical and research environments. It offers a scan speed of 43 s for a 15 mm × 15 mm area at 40× equivalent magnification, alongside a 300-slide capacity. The SG300 delivers high-quality images with a resolution of 0.25 µm/pixel, employing Time Delay Integration (TDI) line scanning and continuous auto-focusing. The system utilizes Philips’ proprietary iSyntax file format and supports various 1D and 2D barcode types. Additionally, the scanner is prepared for multi-layer scanning through its 3D-ready technology [30].

The **VENTANA DP 600** (Mannheim, Germany), developed by Roche, is a high-capacity slide scanner intended for clinical applications. It exhibits a scan time of 60 s for a 15 mm × 15 mm area at 40× magnification, with a total capacity of 240 slides (240 slides arranged in 40 trays of 6). This scanner is notable for its tray-based system, which minimizes slide-handling errors. It offers 20× and 40× scan magnifications with dynamic focus technology, ensuring excellent image quality and accurate color reproduction. It supports diverse slide types, including H&E, IHC, ISH, special stains, and frozen sections, and outputs in BIF, TIF, and DICOM file formats. The system also includes auto-calibration and supports volume scanning with up to 15 layers [33].

The **Grundium Ocus40** (Tampere, Finland) is a distinctive portable scanner primarily intended for research purposes. Despite its compact dimensions (approximately 7 × 7 × 7.5 inches and 3.5 kg), it delivers high-resolution images at 40× magnification, with a resolution of 0.25 µm/pixel. Its scan speed for a 15 mm × 15 mm area is approximately 200 s. It is optimally suited for field use, remote consultations, and educational contexts where portability is a critical factor. It features an intuitive, browser-based interface for convenient scanning, viewing, and sharing of slides, and supports .SVS, .TIFF, and .SZI image formats [41].

Table 4 reveals that the key distinctions among most commercially available brightfield scanners are their resolution, scanning speed, and slide capacity, without considering factors such as operational complexity or cost. For our specific case study, the 3DHISTECH Pannoramic 1000 scanner was selected due to its highest resolution and immediate availability within our facility. Furthermore, its slide magazine system demonstrated compatibility with both the automated stainer and coverslipper employed in our workflow, thereby ensuring seamless integration and operational efficiency. This choice underscores the practical considerations often influencing equipment selection in a laboratory setting.

### 2.4. Collaborative Robotic Arms

We conducted a limited, representative comparison of collaborative robots (cobots) suitable for automating slide transfer. This concise selection is necessary given the wide range of manufacturers and products available, a comprehensive evaluation of which is outside the scope of this paper. Our comparison focuses on robotic arms with 6 or 7 degrees of freedom (DOF) to accommodate the complexity of the task, while also varying in payload capacity and other core features. The specific robots included in this comparison are detailed in Table 5.

The Universal **Robots UR5e** (Odense, Denmark) stands out as a flagship model, widely recognized for its intuitive, graphical programming interface. Its integrated joint force–torque sensors are a key safety feature, allowing the robot to safely interact with humans in shared workspaces [49]. For heavier applications, the **UR20** extends the UR family’s capabilities with a higher payload and extended reach, making it suitable for tasks like palletizing and machine tending that require greater lifting capacity [50].

Similarly, manufacturers like FANUC and ABB have developed robust cobots for industrial use. The FANUC **CRX-10iA** (Tokyo, Japan) is notable for its lightweight and compact design, which allows for flexible deployment, while its collision-stop function and hand-guided teaching simplify operation [51]. **ABB’s GoFa CRB 15000 (Zürich, Switzerland)** focuses on high-payload tasks, utilizing SafeMove software and internal torque sensors to ensure safe operation alongside human workers without the need for traditional safety fences [52].

In a category focused on research and advanced manipulation, the Franka Emika **Franka Research 3** (Zürich, Switzerland) and KUKA **LBR iiwa** (Augsburg, Germany) offer exceptional sensitivity and precision. The direct joint torque control of Franka Research 3 robot makes it an ideal tool for haptic applications and robotics research, supported by seamless Robot Operating System (ROS) integration [53]. The KUKA LBR iiwa is similarly renowned for its high precision and sensitivity, making it a preferred choice for delicate assembly and medical research tasks [54].

Based on the data in Table 5, it is evident that although all cobots are designed for safe human–robot interaction, their specific designs, control methods, and features are optimized for particular applications. Given the properties outlined previously, the UR5e was selected for managing the interactions between the stainer–coverslipper and the scanner. This choice was based on its user-friendly programming interface, robust integrated safety features, and a payload capacity that is well-suited for laboratory equipment, making it the optimal option for this specific task.

## 3. Results

**Figure 1:**
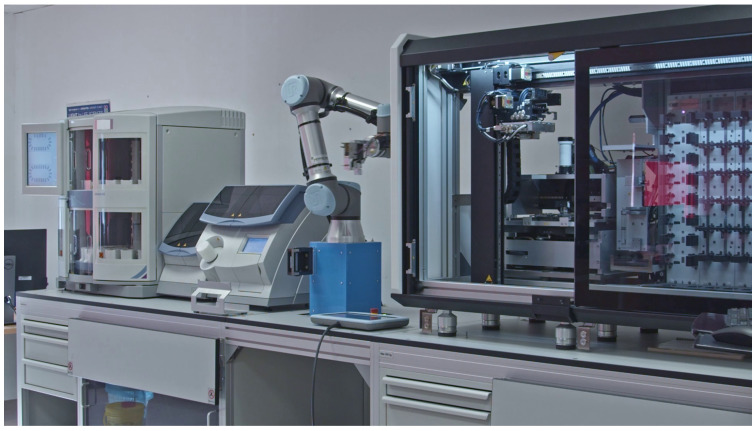
Implementation of an automated pathology workflow, consisting of a stainer, coverslipper, robot arm, and scanner (from left to right on the figure).

The hardware configuration of the automated diagnostic workflow is illustrated in Figure 1. The process begins with a tissue stainer, which applies a predetermined staining protocol to sliced tissue samples on slides. Following this procedure, the stained and dried slides are transferred to the coverslipping section via a conveyor belt mechanism. The coverslipper then applies a cover slip to each prepared slide, which serves to protect the sample from contamination and maintain its integrity. A robotic arm, equipped with a newly developed multi-function, task-specific gripper, is positioned adjacent to the coverslipper. This specialized gripper allows the robot to open the plexiglass door of the coverslipper and extract magazines containing the covered slides. The magazines are then temporarily stored in a holder, where individual slides are removed and fed into a slide scanner. The slide scanner generates high-resolution digital images of the tissue, which are subsequently used for analysis. After scanning, the slides are returned to their magazines, and the magazines are subsequently placed in a local archive. The rendered hardware design of the complete workflow, including all dimensions and distances, created in Solid Edge, is provided in the Figure S1 SolidEdge-Gemini_UR5_P1000_layout_design.png, which is available in the Supplementary Materials.

Our current research builds upon prior methodological contributions to establish a high-level mapping between specific laboratory subtasks and corresponding robot arm movements. This mapping ensures the general feasibility and implementation of the proposed automation framework. The technical foundation for this work is derived from several key advancements: a detailed investigation into magazine detection, position determination, software architecture, and communication protocols [55] (see also Supplementary Materials Video S1: experimental_magazine_detection_phase_1); the development and evaluation of a patented, unique gripper and a system for slide detection and manipulation, which addressed the complexities of handling transparent objects and various slide types [56,57]; and the implementation of a three-point robot calibration method, which was benchmarked against manual and mechanical approaches [58]. The insights gained from these foundational studies enabled the abstraction of tissue preparation processes into a set of generalized robotic movements, thereby providing a versatile framework for automating a range of laboratory procedures.

### 3.1. Custom Gripper

**Figure 2:**
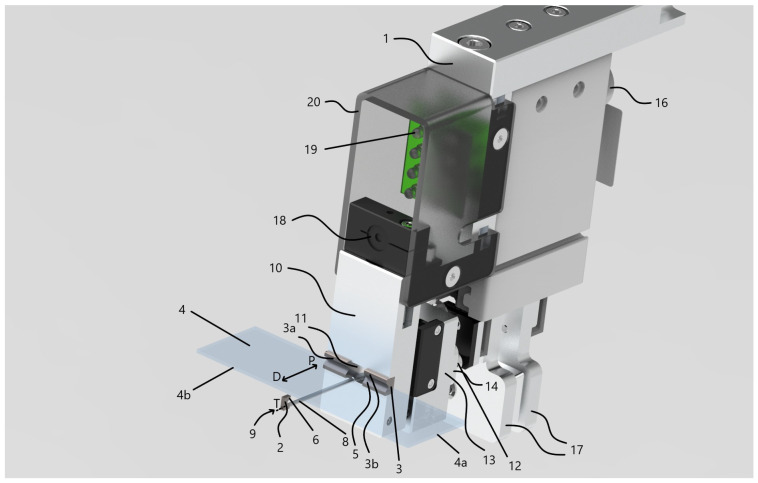
Custom magazine and slide manipulator design created in Solid Edge. Numbered components: 1—robot manipulator assembly, 2—primary gripper, 3—secondary gripper, 3a—first half of the secondary gripper, 3b—second half of the secondary gripper, 4—standard slides, 4a—short edge of a slide, 4b—long edge of a slide, 5—prismatic edge of the secondary gripper (longer), 6—prismatic edge of the primary gripper (shorter), 7—magazine, 8—slide support rod, 9—end of the support rod, 10—metal frame, 11—hole machined into the metal frame, 12—multi-spring members, 13—metal subframe for the springs, 14—translational unit of the actuator, 15—rotational unit of the actuator, 16—robotic arm, 17—magazine clamps, 18—miniature endoscope camera, 19—LED lighting module, 20—plastic diffusing cover.

We have found that no commercially available robotic arm tool currently exists that is capable of manipulating both magazines and the individual slides within them, which could perform tasks such as the removal of the slides from one magazine, precise positioning, and the reinsertion into another magazine. Additionally, none of the existing systems support the direct transfer of either the complete magazines or the individual slides to an external device. Therefore, it was necessary to design and fabricate a custom manipulator to fulfill these requirements. The rendered design of the final manipulator, which has been patented [56], is shown in Figure 2.

The overall manipulator assembly 1 is built on a robust metal frame 10, which provides the structural foundation for attachment to a robotic arm. The frame is designed to mount onto the standard robot flange interface (ISO 9409-1-50-4-M6) [59,60]. This standardized connection is available on robot arms from various manufacturers, which ensures that the mechanical interface of the manipulator is compatible with multiple robot types. Consequently, the manipulator can be applied to any robotic system that complies with this standard.

The manipulator 1 is equipped with two opposing slide-gripping units—referred to as the primary 2 and secondary 3 grippers. Both are implemented as V-shaped prismatic jaws, each with angled stems. While the included angle between the stems is typically 90 degrees, alternative angles commonly used in prismatic jaw designs are also feasible. The jaws of both gripping components 2 and 3 are fabricated from a hard, wear-resistant metal—specifically tungsten carbide, whose chemical composition is tungsten and carbon (WC). WC was selected because these parts of the tool come into direct contact with the edges of the glass slides during each gripping operation, which can lead to wear over time. Therefore, a highly wear-resistant material was required. The dimensions of the jaws are optimized to securely engage the long sides 4b of standard slides 4. Notably, the secondary gripping unit 3 features a longer prism edge 5 compared to the edge of the primary unit 6, making it wider. The primary gripping unit 2, by contrast, is designed to be narrow enough to fit between two adjacent slides 4 within a magazine 7, by means of its prism edge 6 being shorter than the spacing distance H between neighboring slides. In terms of width, the prismatic edge of the primary gripping unit 2 typically measures between 1–2 mm, while that of the secondary unit 3 ranges from approximately 10–50 mm.

The primary gripping unit 2 is attached to one end 9 of a support rod 8, which is capable of rotational movement about its longitudinal axis T, allowing switching between two defined positions. The support rod 8 is a circular cross-section with its diameter kept not greater than the length of the first prismatic edge 6. When the support rod 8 is in its initial position, the V-shaped gripping surfaces of units 2 and 3 directly face one another, ensuring that the slide 4 being held is not subjected to any rotational torque. Rotating the rod 8 by 90 degrees places it in its second position, thereby aligning the primary gripping unit perpendicularly relative to its orientation in the first position.

The secondary gripping unit 3—comprising two separate prismatic jaws 3a and 3b—is fixed to the frame 10, while the support rod 8 passes through a hole 11 machined into the frame. The two-part construction of the gripping unit 3 eliminates the need to drill through a single solid carbide block, which offers a significant advantage in terms of manufacturability and tool longevity when working with hard materials like tungsten carbide.

In the final embodiment, three multi-spring members 12 are incorporated, implemented as coil springs. Each spring is anchored at one end to the main frame 10, while the opposite end is attached to a rigid, guided sub-frame 13. Although coil springs are used in this configuration, alternative elastic components may also be substituted as needed. Axial movement of the support rod 8 along direction P is actuated by the set of spring members 12, which apply a preloaded force to maintain slide retention. The clamping force can be fine-tuned by adjusting the design parameters of the spring members 12, thereby ensuring consistent and optimal holding pressure on the slides 4 by the gripping units 2 and 3 during manipulation.

The gripper assembly integrates two actuation mechanisms: a translational unit 14 for moving the support rod 8 linearly along its longitudinal axis T, and a rotational unit 15 responsible for rotating the support rod 8 around the same axis. The moving actuator 14 may be realized using any device capable of producing straight-line motion—such as a rail-guided carriage or a track-based system. The rotational actuator 15, mounted on the distal end of the support rod 8, is responsible for rotating the rod by a specific angle and maintaining it in position. This actuator may be implemented using a stepper motor or a DC servo motor. In our design, the position control of the rotational actuator is implemented using a stepper motor operating in an open-loop configuration. The motor drives the support rod around its longitudinal axis by executing discrete rotational steps corresponding to predefined angular displacements ($90^{{}^{\circ}}$ for approaching and $0^{{}^{\circ}}$ for grabbing the slide). System calibration is achieved through a homing procedure, in which the mechanism is driven to a mechanical stop serving as the reference (zero) position. After homing, all subsequent angular positions are determined by precise step counting, ensuring repeatable angular positioning. The operational state of the system is verified by a Hall-effect magnetic field sensor, which provides binary confirmation of the rod’s position (ON/OFF) at key operational states. This hybrid control strategy offers sufficient precision and robustness for repeated slide manipulation without the need for complex closed-loop feedback electronics. Furthermore, the power transmission to the rod can also be achieved through various mechanisms, including rack-and-pinion systems, lead screws (spindles), or belt drives.

The magazine clamps 17, mounted on the robotic arm 16, are positioned in direct contact with the spring frame 13. As a result, the spring members 12 continuously press the frame 13 against the magazine clamps 17. This action causes the clamps 17 to open, allowing the magazine hook to be securely engaged. To close the clamps, the spring frame must be disengaged from the clamps. This mechanism enables the magazine gripper to grasp and then manipulate entire magazines in arbitrary 3D positions within the robot base coordinate system. Additionally, when the magazine clamps 17 are opened, they exert force on the frame 13, pushing it along direction D. This motion is transmitted to the support rod 8, which moves accordingly along its longitudinal axis T in the same direction. Through this mechanism, individual slide 4 can also be reliably grabbed and relocated by the manipulator, maintaining full freedom of movement within the three-dimensional workspace of the robot.

A constraint of the system is that the magazines must have a feature, such as a handle or hook, that a custom gripper can latch onto. The specific design of this feature can vary without significantly altering the gripping procedure. For slides, the custom manipulator requires them to be 1 mm thick and 25 mm wide. These dimensions are not highly restrictive, as they correspond to the standard sizes of most currently used slides. However, if deviation from these parameters is desired, appropriate modifications to the manipulator design must be implemented accordingly.

### 3.2. Applied Sensors

The applied cobot also incorporates several sensing components that play a crucial role in ensuring safe and efficient human–robot collaboration. These sensors enable the system to monitor its environment, verify correct operation during each manipulation step, and ensure compliance with safety standards. The integration of such sensing elements is essential not only for preventing equipment damage and ensuring human safety but also for maintaining the precision and reliability required in automated laboratory handling tasks. This section provides details about the sensors integrated into the workflow.

1–6. The cobot used in this study is equipped with integrated joint force–torque sensors, which are key safety features allowing the robot to safely interact with humans in shared workspaces. These six sensors enable the detection of any collisions with objects, laboratory equipment, or human operators within the 3D workspace. In our setup, potential collision scenarios include accidental contact with the equipment due to, for instance, a misaligned magazine placement, an excessive gripping force that could result in slide breakage, or unintended contact with a laboratory technician entering the workspace. To prevent such events, predefined force thresholds are implemented in the control software of the robot. When the measured joint forces exceed the preset limit, the robot immediately suspends the current operation. In our case, the safety force threshold was conservatively set to 15 N, which is well below the typical slide fracture limit (≈20–30 N) and far under the contact force considered safe for human–robot interaction according to ISO/TS 15066 (65–660 N) [61]. This ensures the safety of humans, equipment, and tools, as well as the integrity of the most sensitive element in the system—the fragile glass slides being handled.
7. For magazine and slide detection, a digital, complementary metal oxide semiconductor (CMOS) UVC Signal miniature endoscope camera (model type: RD-V31110RL-77-03) manufactured by MISUMI [62], with an aperture setting of f=2, was fixed on the frame unit of the manipulator horizontally. (Note: In optics, the *f*-number represents the ratio between the focal length and the aperture diameter in optical systems.) Since environmental conditions—such as variations in ambient light throughout the day—can introduce visual noise into the captured images [63,64], additional illumination was integrated to stabilize image quality. To mitigate these effects, a vertically oriented 2 × 4 in-line LED lighting module was installed on the same frame, directed toward the camera. In addition, a plastic diffusing cover was positioned around the LEDs to promote uniform light dispersion, enhancing illumination consistency and thus improving the robustness of image processing.
8. For magazine manipulation, a magnetic field sensor (model type: MFS02-K_KHC-P2_PNP) manufactured by Zimmer Group was integrated into the housing of the magazine gripper [65]. The sensor detects the position of the internal sliding element equipped with a small magnet and provides two distinct switching points corresponding to the fully open and fully closed gripper states of the magazine clamps. This setup enables reliable real-time monitoring of the gripping process. The given feedback was used as a safety control mechanism to verify proper grabbing operation and to prevent excessive force application that could cracking to the brittle polymer magazines.
9. For slide grabbing, a Hall-effect magnetic field sensor (model type: Infineon TLE49x5L) manufactured by Infineon Technologies AG was integrated into the slide gripper mechanism [66]. The sensor operates based on the Hall-effect, detecting the magnetic field generated by a small permanent magnet mounted on the rotating slide support rod and generating a digital ON/OFF signal depending on its angular position. When the rod rotated to the required $90^{{}^{\circ}}$ position, the sensor output switched to the ON state, confirming that the rod can enter the narrow gap between adjacent slides inside the magazine. Upon rotating the rod to its initial $0^{{}^{\circ}}$ position, the sensor switched back to the OFF state, indicating that the rod was realigned to support and safely extract the selected slide from the slide. Its key advantages are the high reliability and contactless operation, ensuring wear-free detection during the repeated gripping of the edges of the slides.
10. For slide rotation, an optical gate sensor (model type: TCST1230) manufactured by Vishay Semiconductors was integrated into the slide layout rotator of the scanner [67]. The sensor operates based on the interruption of an infrared light beam between the emitter and the receiver pair. In this setup, the optical gate was positioned at both end stops of the rotation axis to detect the two limit positions ($0^{{}^{\circ}}$ and $90^{{}^{\circ}}$). During operation, when the layout rotator entered one of these limit positions, a small flag mounted on the shaft interrupted the light beam, triggering a digital signal. This feedback was used by the control system of the robotic arm to verify that the rotation mechanism reached its intended position and to prevent overtravel or step loss of the motor. As a result, the optical gate provided a simple yet reliable method for ensuring accurate and safe slide rotation alignment.

### 3.3. Mapping Manual User Interactions of Coverslipper to Robot Movements

**Figure 3:**
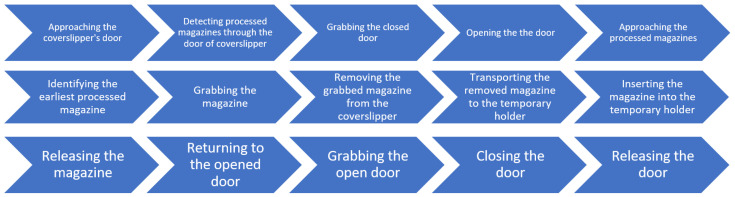
Overview of the magazine manipulation workflow process.

**Figure 4:**
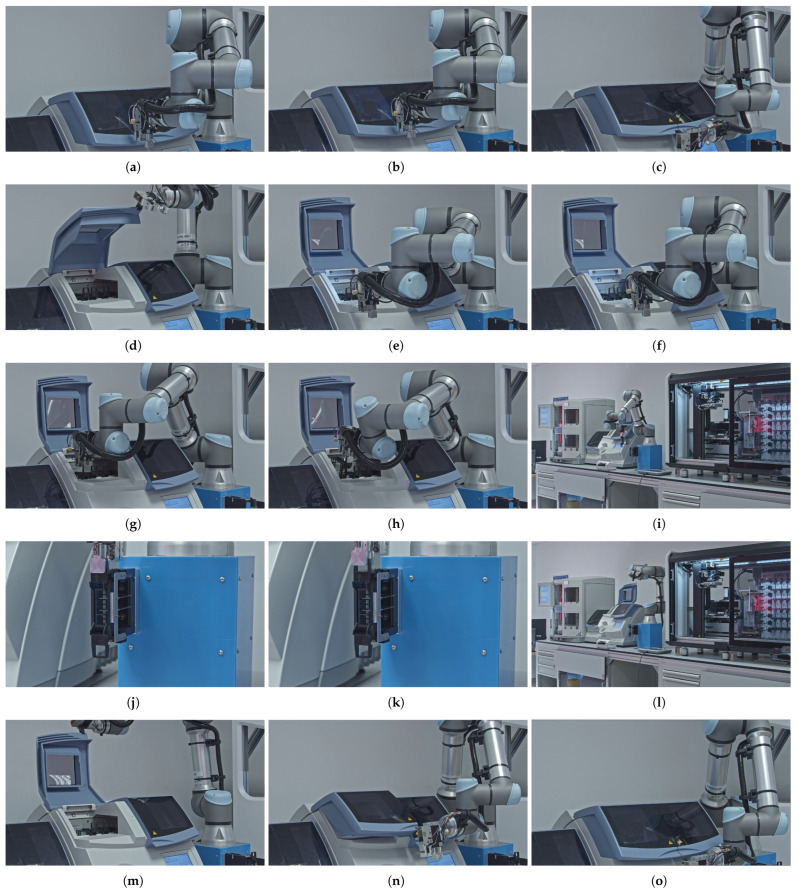
Detailed robotic movements required for transporting the magazines from the coverslipper to the scanner. (**a**) Approaching the coverslippers’s door. (**b**) Detecting the processed magazines though the door. (**c**) Grabbing the closed door. (**d**) Opening the door. (**e**) Approaching to the processed magazines. (**f**) Identifying the earliest processed magazine. (**g**) Grabbing a magazine. (**h**) Removing a grabbed magazine from the coverslipper. (**i**) Transporting a removed magazine to the temporary holder. (**j**) Inserting a magazine into the temporary holder. (**k**) Releasing a magazine. (**l**) Returning to the opened door. (**m**) Grabbing the open door. (**n**) Closing the door. (**o**) Releasing the door.

An overview of the magazine manipulation workflow process is provided in Figure 3, highlighting the sequence of the operations. The sequence steps in the diagram are aligned with the elements presented in the corresponding subfigures in Figure 4 that illustrate the key stages of an automated magazine manipulation performed by the robotic arm. Starting with the arm approaching the coverslipper’s door (a), then the system detects the presence of processed magazines (b). After that, it grasps and opens the door (c, d) to gain access to the magazines (e). Subsequently, the vision-based system identifies the earliest processed magazine from the stack (f), allowing the arm to grab it (g) and remove it from the coverslipper (h). The retrieved magazine is then transported to a temporary magazine holder of the robot (i), where it is thereafter accurately inserted (j) and released (k). Finally, the robot returns to the coverslipper’s door (l), grasps (m), closes (n), and releases it (o), completing a full cycle of magazine retrieval.

Based on the analysis of the program code related to the subtasks, Table 6 presents a detailed decomposition of the robot movements, mapping the sub-steps of automated magazine manipulation to the required degrees of freedom (DOFs) of the robotic arm. The columns Rot. X, Rot. Y, and Rot. Z and Trans. X, Trans. Y, and Trans. Z indicate the rotational and translational movements, respectively, with a plus sign (+) denoting an active DOF for a given step. The TCP manipulator column specifies which element of the manipulator (e.g., LED, camera, image processing, or specific gripper) is actively used during each operation. Additionally, both the door and the magazine grippers contribute an additional translational DOF each, which is added in parallel at the end of the robot’s existing kinematic chain—one on each side of the manipulator. The Sum column quantifies the number of DOF required for each individual sub-step, while the Cumulative amount column tracks the number of unique DOF utilized so far during the entire process. Thus, the final Cumulative amount represents the total DOF necessary to complete the entire automated magazine handling task. For two steps, where the symbol “↔” appears next to the step identifier (letter label), it indicates that the motion sequence involves the same combination of movements but executed in opposite directions (for example, Opening vs. Closing a door). Additionally, the symbol “=” at the identifier denotes motion steps that are equivalent in terms of movement composition but belong to different workflows.

### 3.4. Mapping Manual User Interactions of Tissue Scanner to Robot Movements

**Figure 5:**
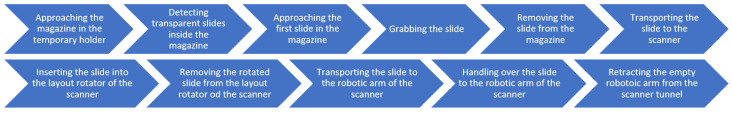
Overview of the slide manipulation workflow process.

**Figure 6:**
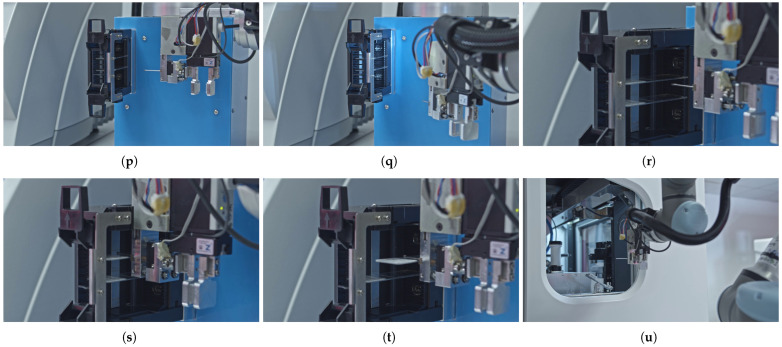

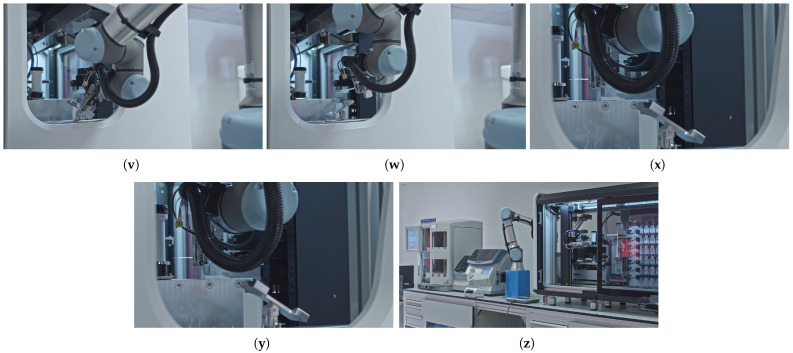
Detailed robotic movements required for transporting the slides between the magazine holder and the scanner. (**p**) Approaching the magazine in the temporary holder from the side. (**q**) Detecting a transparent slide inside a magazine. (**r**) Approaching a detected slide. (**s**) Grabbing a slide. (**t**) Removing a slide from a magazine. (**u**) Transporting a slide to the robotic arm of the scanner. (**v**) Inserting a slide into the scanner layout rotator. (**w**) Removing a (rotated) slide from the scanner layout rotator. (**x**) Transporting a slide to the robotic arm of the scanner. (**y**) Handling over a slide to the robotic arm of the scanner. (**z**) Retracting the (empty) robotic arm from the scanner tunnel.

Figure 5 provides a comprehensive overview of the slide manipulation workflow process, highlighting the sequence of the operations. The sequence steps in the diagram are aligned with the elements illustrated in the corresponding subfigures in Figure 6 that present the key stages of an automated slide manipulation during the robot operation. The process begins with the arm approaching the magazine previously inserted in the temporary holder (p) and then detecting the transparent slides within it by computer vision and image processing algorithm (q). After that, the robot approaches (s), grabs (t), and removes the detected slides from the magazine (t) one by one. In the subsequent step, the next slide in line is transported (u) and inserted into a scanner layout rotator (v). After rotation, the slide is removed (w) and transported toward the scanner’s own robotic arm (x). The slide is handed over to the scanner (y), and the empty arm retracts from the scanner through the designated scanner tunnel (z). Once the scanning of the slide is completed, the scanner software sends a command to the robotic arm’s control box to retrieve the next pre-detected slide. To execute this, the robotic arm repeats the tasks described above (r–z) for each individual slide, in a loop, until all detected slides from the magazine located in the temporary holder have been sequentially transferred. At this point, the processed samples are stored in dedicated magazines of the scanner, which differs from the input one, and are ready for the next stage of the pathological workflow.

Following the same methodology as outlined in Section 3.3, Table 7 presents a detailed decomposition of the robot movements, mapping the subtasks of automated slide manipulation to the required DOF of the robotic arm. The column definitions, the active Tool Center Point (TCP) manipulator, and the Sum and Cumulative amounts of the DOFs are consistent with the principles outlined in the previous section. Furthermore, the applied slide gripper introduces two additional degrees of freedom—one rotational and one translational—into the robot’s existing kinematic chain, as indicated in the TCP column.

### 3.5. Mapping Manual Handling of Processed Slides and Magazines to Robot Movements

**Figure 7:**
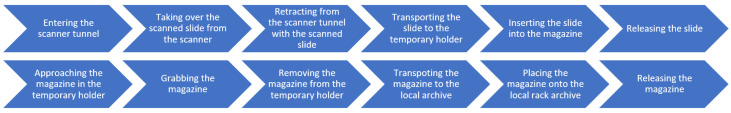
Overview of local archiving workflow process.

**Figure 8:**
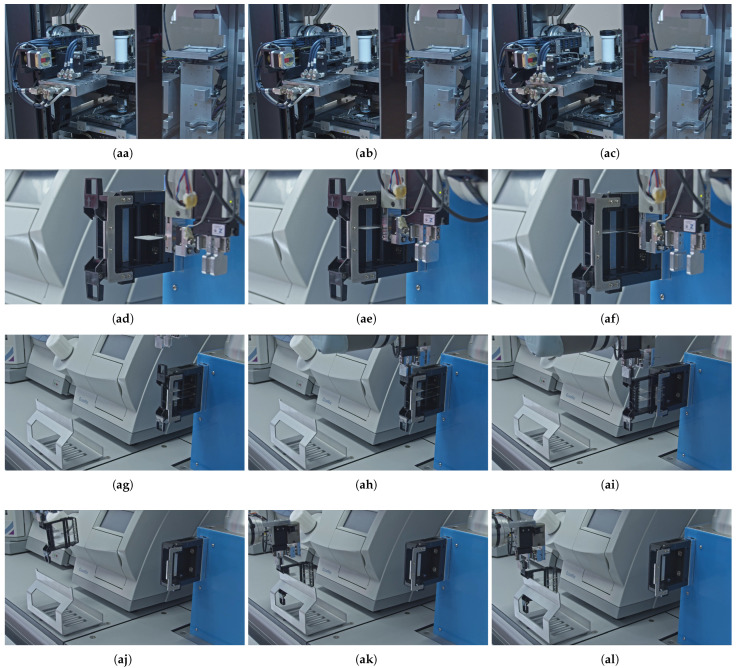
Detailed robotic movements required for the local archiving of the processed slides and magazines. (**aa**) Entering the robotic arm to the scanner tunnel. (**ab**) Taking over a (scanned) slide from the robotic arm of the scanner. (**ac**) Retracting the robotic arm with a (scanned) slide from the scanner tunnel. (**ad**) Transporting a slide to the temporary holder. (**ae**) Inserting a slide to a magazine. (**af**) Releasing a slide. (**ag**) Approaching to a magazine in temporary holder from the front. (**ah**) Grabbing a magazine. (**ai**) Removing a magazine from the temporary holder. (**aj**) Transporting a magazine to the local archive. (**ak**) Placing a magazine onto the local rack archive. (**al**) Releasing a magazine.

To address both the alternative handling of processed tissue slides and the removal of used magazines from the interaction point of the workstation, we developed dedicated automated solutions. In cases where the user wishes to retrieve the scanned slides for further individual (re)use—such as for additional staining procedures or local storage—a specific pre-programmed robotic procedure can be initiated, the overall sequence of which is outlined in Figure 7. This overview highlights the main stages of the local archiving workflow for the processed slides and magazines, which are further detailed in the corresponding subfigures of Figure 8 that visualize the key steps of the automated process performed by the robotic arm, which operates as follows. Once the scanner software sends a signal to the robotic arm’s control box indicating that the given slide has been processed, the robotic arm, prior to introducing a new slide according to the steps described in Section 3.4, first re-enters the scanner tunnel with an empty manipulator (aa) and takes over the previously scanned slide from the internal robotic arm of the scanner (ab). Then it retracts with the slide (ac) and transports it back to its original slot within the corresponding magazine located in the temporary holder (ad). Following that, the robotic arm inserts the slide into the magazine (ae) and releases it (af) at its original position. Only then does the robot proceed with introducing the next slide into the scanner.

Regardless of whether the user intends to retain the processed slide for further use or allows it to be stored in the internal storage of the scanner, the original magazines that have already been utilized must be removed from the interaction point of the active workflow. This step is essential to enable the processing of the next magazine containing newly stained slides. In both scenarios, the robotic arm executes the same standardized removal procedure. To do so, the robot approaches the temporary holder now from the front side (ag). It then grasps the used magazine (ah), removes it from the holder (ai), and transports it to the local archive rack (aj). Finally, the robot places (ak) and releases (al) the magazine onto the storage rack, thereby concluding the full operational cycle.

Consistently, following the same methodology used for the coverslipper and scanner interactions, the decomposition of the robot movements for the archiving steps is presented in Table 8. The table columns, such as the active Tool Center Point (TCP) manipulator, the sum of DOF for each step, and the cumulative amount of unique DOF, are defined consistently with the previous tables. A dashed line marks the boundary up to which steps are executed only if the user intends to retain the processed slide for further reuse. Robot movements listed beyond this line correspond to the removal of the processed magazines from the workflow either way.

**Figure 9:**
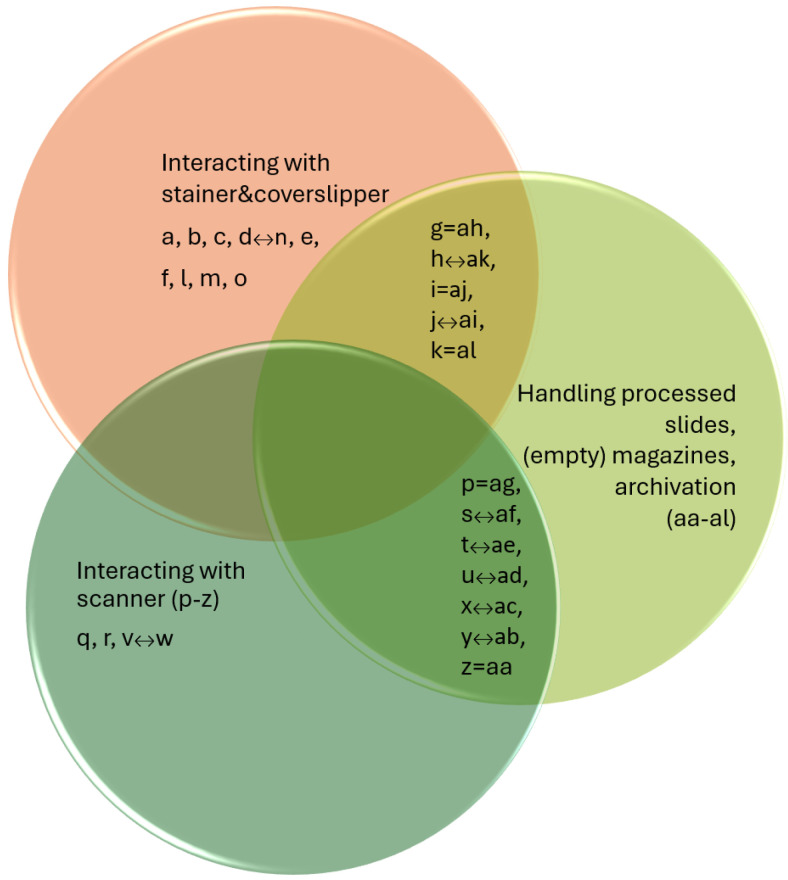
Venn diagram of robotic movements for the examined laboratory tasks, highlighting the overlapping steps of the automation.

In addition, Figure 9 summarizes, in the form of a Venn diagram, all the laboratory work steps examined in Section 3.3, Section 3.4 and Section 3.5. It also correlates and identifies the overlap between the robotic movements of the tasks performed in the different phases, thereby allowing for the determination of the minimum number of steps per phase and in total.

### 3.6. Workflow Testing

To provide a comprehensive evaluation of the developed robotic workflow, both qualitative and quantitative performance testing was conducted across multiple phases, addressing individual components, their integration, as well as full-system operation under realistic conditions. The testing strategy aimed to generate key performance indicators (KPIs), such as total cycle time, efficiency improvements relative to manual operation, and system reliability, thereby demonstrating the advantages of the proposed system.

Testing began with unit-level evaluations of the hardware and software components to ensure that each function or method produced the expected output. Test cases included verification of the configuration module, gripper and camera modules, Python-based (v3.7.6) image processing routines, magazine detection, slide detection, calculation of 3D object positions from images, and robot manipulation capabilities such as opening the coverslipper door, and grasping magazines and slides. Each individual workflow step, as detailed in the Results Section tables, was systematically tested. In total, over 300 unit test cases were executed, providing a strong foundation for subsequent integration testing.

This was followed by integration testing, focusing on the correct communication and data exchange between modules required to execute the workflow. The tested key interactions included the camera module with the calibration module, the image processing module with the robot controller, the robot controller with robotic arm hardware, and the robot controller with scanner software. Each integration cycle was repeated 40–50 times to verify stable and consistent performance across interacting components.

System-level testing comprised both functional and non-functional evaluations. Functional tests were conducted using smoke, sanity, and regression test cycles. Smoke tests assessed whether the build was sufficiently stable to allow further testing by confirming that the system’s crucial functions could execute. Sanity tests verified that newly implemented features functioned as intended in the current build. Regression tests ensured that modifications did not introduce undesired side effects to previously verified functionalities. These functional tests were executed 5–10 times per feature.

Non-functional tests included capacity testing to determine the speed and the maximum number of transactions the system could handle without performance degradation, endurance testing to evaluate system stability over one week of continuous operation under typical load conditions, and reliability testing to assess consistent system performance under maximum slide handling capacity, corresponding to 1000 slides (around 50 magazines). Furthermore, a comparison with the manual mode was included, in which the average time required for each individual task was evaluated between robot and manual operation, as shown in Table 9.

Following system-level tests, acceptance testing was performed, incorporating both verification and validation procedures. Verification confirmed that the system’s end-to-end behavior met project specifications in-house. Validation ensured that the system fulfilled user and client expectations. Eight independent scenarios were executed, including on-site trials by potential clients (three cases), as well as evaluations at new installation sites in Hungary (two cases), the USA (one case), and Switzerland (one case). Feedback from these trials informed final refinements of the validated system, which was subsequently delivered to an industrial partner in the USA.

An operational sequence of this automated system during its workflow testing is documented in the Video S2, which is available in the Supplementary Materials, while the key performance indicators resulting from these tests are summarized in Table 10, including the task capacity, object detection precision, positional accuracy, total cycle time, and cost, thereby quantitatively demonstrating the achieved efficiency of the system.

The testing phases yielded several representative failure cases, which were addressed to enhance system robustness and reliability. The following list presents the five most significant issues encountered during testing along with the solutions implemented to mitigate them.

1. Ambient light variations: Changes in environmental lighting introduced visual noise, negatively affecting image capture. This issue was mitigated by installing a 2 × 4 LED illumination module over the camera with with a diffusing cover, stabilizing image quality and improving the robustness of image processing.
2. Magazine detection interference: Direct overhead lighting caused glare on the black plexiglass door of the coverslipper, impairing magazine detection. The solution involved slightly tilting the camera during detection to avoid reflected light entering the lens.
3. Incorrect magazine positioning: Misalignment during gripping could crack magazines. Conical magazine clamps were implemented, providing an additional 1 mm tolerance to ensure secure and safe magazine capture.
4. Slide grip failures due to size or damage: Broken or irregularly sized slides could slip or break. Sensor-based monitoring of gripping force was employed to confirm successful grasping.
5. Slide adhesion after staining: Improperly covered or sticky slide edges caused adherence to the magazine holder. The solution involved calibrating the staining module during robot integration, followed by monthly recalibration, which mitigated these issues.

## 4. Discussion

This study involved a comparative analysis of commercially available automated tissue stainers, coverslippers, and digital slide scanners. Furthermore, we have also reviewed a range of industrial and medical collaborative robots from various manufacturers, identifying those suitable for integration into a fully automated pathology workflow.

To implement the proposed concept, we evaluated the key features of staining devices, including their supported protocols, slide rack capacity, maximum number of racks, throughput, special features, system compatibility, and local availability. This assessment led to the selection of the Gemini AS Automated Slide Stainer. For coverslipping instruments, we considered criteria such as covering material, drying time, maximum throughput, and system compatibility, which resulted in the acquisition of the ClearVue Coverslipper. In parallel, digital slide scanners were compared based on their resolution, numeric aperture, scanning speed, slide capacity, and intended use. This evaluation led to the choice of the Pannoramic 1000. To integrate the workflow, we assessed collaborative robots according to their primary application, degrees of freedom, payload capacity, control method, integrated sensors, and notable features. Based on this assessment, the UR5e robotic arm was selected to connect and automate the devices within the workflow.

During the implementation, we empirically determined the minimal set of manual handling steps required for processing tissue samples mounted on slides and stored in magazines, as part of the integration of staining, coverslipping, and scanning devices. For the combined staining and coverslipping equipment, this process consists of 15 distinct steps, as detailed in the subfigures of Figure 4. Each of these steps was mapped to specific robotic movements and corresponding manipulator modules. This mapping allowed us to determine the minimum degrees of freedom required for the robot to perform these tasks, both individually and cumulatively, as shown in Table 6. We identified that to replace the manual user interaction with the integrated tissue stainer and coverslipper, the robot requires 5 of its 6 DOFs. A custom manipulator, mounted on the robot’s flange, was also necessary. This manipulator must incorporate a camera system for computer vision and image processing, an LED, a gripper for the coverslipper door, and a magazine gripper. These two additional grippers each provide one translational DOF, which are added in parallel to the robot’s existing kinematic chain. Consequently, the robot requires a total of 7 DOFs to complete these operations. However, since the two grippers are not used simultaneously in any single task, only 6 DOFs are actively engaged at any given time.

A similar approach was used to determine the minimum number of manual handling steps required for processing slide-mounted tissue samples with digital slide scanners. We identified a set of 11 discrete steps for this process, which are detailed in the subfigures of Figure 6. Each of these steps was correlated with specific robotic movement types and the necessary manipulator modules. This analysis allowed us to ascertain the minimum degrees of freedom (DOFs) needed for the robot to execute each task individually and cumulatively, as presented in Table 7. The mapping revealed that the robot requires 5 of its 6 native DOFs to automate the user interactions with the digital slide scanner. Furthermore, a custom manipulator, fixed to the robot’s flange, is essential. This manipulator must be equipped with an LED and camera system for computer vision, in addition to a slide gripper. The slide gripper contributes 2 additional DOFs—one rotational and one translational—to the robot’s existing kinematic chain. Consequently, the robot requires a total of 7 DOFs to perform these tasks autonomously. Since the two DOF of the slide gripper are always used in conjunction, all 7 DOFs will be simultaneously engaged during operation.

Following the same empirical methodology, we defined the minimum manual steps required for extracting processed slides and magazines from the workflow and storing them in a local archive. This process comprises 12 distinct steps, which are detailed in the subfigures of Figure 8. We then mapped each of these steps to specific robot arm movements and the necessary manipulator modules, as shown in Table 8. This mapping revealed that the robot needs 5 of its 6 native degrees of freedom to automate the interaction with the digital slide scanner. The custom manipulator, which is mounted on the robot’s flange, must include both a slide gripper and a magazine gripper. The slide gripper contributes 2 DOFs, while the magazine gripper adds 1 DOF, expanding the robot’s serial chain in a parallel configuration. Consequently, the total DOFs required to complete these tasks is 8. However, because the two grippers are never used at the same time, a maximum of 7 DOFs will be simultaneously active during any given task.

By consolidating all three cases, it was determined that in order for the robotic arm to perform all the examined functionalities, a total of 9 DOFs is required. Of these, 5 DOFs (translation: X, Y, Z; rotation: X, Z) belong to the robot itself, while 4 DOFs (3× translation, 1× rotation) are associated with the grippers of the custom manipulator. We compared the minimal set of manual handling steps for each functionality, which is represented in Figure 9. There is no overlap between the operations of the stainer and coverslipper and the scanner; however, when extracting processed slides and magazines from the workflow for local archiving, both share significant overlap. For the stainer and coverslipper, there are 3 movements that directly correspond, and 2 movements with identical motion combinations but opposite orientations (e.g., insert magazine into temporary holder/remove magazine from holder). For the scanner interactions, 2 movements correspond directly, and 5 movements share the same motion combinations but with opposite orientations. Taking these into account, the overall complexity of this automatic workflow requires training the robotic arm on a minimum of 24 distinct laboratory tasks.

Quantitative performance analysis revealed that each automated laboratory task was executed within seconds, confirming that the robotic system introduces no operational bottlenecks. Instead, it provides a substantial time efficiency gain over manual operation by enabling continuous magazine and slide manipulation between the stainer, coverslipper, and scanner—entirely without human intervention. This efficiency further enables the parallel use of additional stainers, coverslippers, and scanners, thereby increasing the overall throughput of the laboratory. The system can operate continuously 24/7, effectively replacing the manual workload of three laboratory shifts while maintaining consistent quality across the consecutively processed slides. Image-based object detection maintained an accuracy within 1–2 pixels, while 3D position estimation from images achieved sub-millimeter precision. The comprehensive test program—comprising over 300 unit tests, 40–50 integration cycles, and the handling of several hundred magazines and thousands of slides—validated both hardware and software robustness. Typical failure cases were analyzed and resolved through targeted design refinements, resulting in improved illumination uniformity, enhanced sensory gripping control, and long-term calibration stability.

Regarding generalizability, the custom manipulator was designed to mount onto the standard robot flange interface (ISO 9409-1-50-4-M6). This standardized connection is available on robot arms from various manufacturers, which ensures that the mechanical interface of the manipulator is compatible with multiple robot types. Consequently, the manipulator can be applied to any robotic system that complies with this standard. At the DOF level, this means that the additional 4 DOFs of the manipulator can be seamlessly integrated in series as an extension of the existing kinematic chain of such robots.

The primary limitations of this implementation originate from the necessity of designing a custom manipulator. This component must be equipped with an LED and a camera to facilitate object detection using computer vision and digital image processing. Additionally, it must feature specific grippers capable of grasping equipment doors, magazines, and slides based on the calculated positions. As no suitable commercial manipulator was available, we developed a proprietary solution. This development represents an improvement over existing mass-production solutions.

A constraint of the system is that the magazines must have a feature, such as a handle or hook, that our custom gripper can latch onto. The specific design of this feature can vary without significantly altering the gripping procedure. For slides, the custom manipulator requires them to be 1 mm thick and 25 mm wide. These dimensions are not highly restrictive, as they correspond to the standard sizes of most currently used slides. The system has also been tested with various slide types, including those with a membrane. Should slides of different dimensions be used, the slide gripper would need to be adjusted to accommodate the new sizes.

Regarding workspace limitations, the automated workflow equipment was arranged in a linear configuration without any tilting or rotation. The total dimensions of the setup are 3866 mm in length, 1921 mm in height, and 1138.5 mm in depth. For safety, a 1 m safety zone, separated by a cordon, is required in front of the workflow to account for the robot movement. An additional 0.5 m of space is needed behind the setup for power supplies, cable management, and to facilitate maintenance. Consequently, a laboratory space of adequate size is essential to accommodate this workflow.

A potential future enhancement for the design involves a circular layout, where the robotic arm is centrally located to serve the surrounding equipment. The primary physical constraint of this configuration is the workspace of the robot arm, which, for our selected model, is a sphere with a 1900 mm diameter, extending 850 mm from its base joint. The pathological devices must be arranged within this space to be accessible to the stationary robotic arm.

## 5. Conclusions

This study involved a comprehensive comparative analysis of commercially available automated tissue stainers, coverslippers, and digital slide scanners, alongside a review of industrial and medical collaborative robots from various manufacturers. Based on this analysis, suitable devices and a UR5e robotic arm with a custom-designed manipulator were selected to implement a fully automated pathology workflow. By consolidating all magazine and slide manipulation tasks, including archiving operations, it was determined that a minimum of 24 distinct laboratory tasks must be automated. To execute all required functionalities, the robotic arm requires a total of 9 degrees of freedom (DOFs): 5 DOFs from the robot itself (translation: x, y, z; rotation: x, z) and 4 additional DOFs incorporated into the gripper units of the custom manipulator. The manipulator mounts to the robot flange using the standardized ISO 9409-1-50-4-M6 interface, ensuring compatibility with multiple robotic platforms and seamless integration of the additional DOF into the existing kinematic chain.

Quantitative testing confirmed that each task can be executed within seconds, reflecting the high operational speed of the system and enabling continuous and parallel manipulation of magazines and slides between the stainer-coverslipper and scanner, entirely without human intervention. The system achieved image-based object detection with 1–2-pixel precision and sub-millimeter 3D positioning accuracy, operating in real time under open-air conditions with varying ambient light. Its 24/7 operational capability substantially improve throughput, effectively replacing three manual laboratory shifts while maintaining consistent performance.

In future work, the workflow will be extended to include automated extraction of slides from the local scanner archive and their transport to a global archive, potentially using a mobile robotic arm or tracked system, further minimizing the spatial footprint required within laboratory environments.

## Figures and Tables

**Table 1:** Overview of automated tissue stainers, ordered by their maximum stainable slides per hour to indicate potential throughput. In column 3, “station”, “rack”, “magazine”, and “position” are used interchangeably to refer to slide-holding components, despite variations in manufacturer terminology and mechanical design.

Stainer Model	Vendor	Maximum Number of Magazines	Slide Capacity of Magazines [slides/mgzn.]	Maximum Throughput [slides/hour]	Supported Staining Protocols	Special Features	System Compatibility
Tissue-Tek Prisma Plus Automated Slide Stainer	Sakura Finetek Co. (Tokyo, Japan)	Up to 3 start and 5 end stations	10 or 60	530	H&E, special stains	High-volume throughput, parallel runs, barcode reader, Tissue-Tek iSupport	Tissue-Tek Film Coverslipper or Tissue-Tek Glas g2 Coverslipper
HistoCore SPECTRA ST Stainer	Leica Biosystems (Nussloch, Germany)	12 racks	30 (5 for special stains)	360	H&E, special stains	Continuous loading, simultaneous protocols, integrated fume control	Leica HistoCore SPECTRA CV Coverslipper
Gemini AS Automated Slide Stainer	Epredia, a Thermo Fisher Scientific Brand (Kalamazoo, Michigan, USA)	10–15 magazines	20	200–300	H&E, special stains	Compact, dual-level design, STAT feature	ClearVue Coverslipper
JQ-DS200 Slide Stainer and Coverslipper	Guangdong Jinquan Medical Technology Co. (Shenzhen, Guangdong, China)	7 buffer and 2 input and output positions	20	200	H&E, special stains	No xylene/alcohol, priority stain function, barcode reader	Staining and covering in one system

**Table 2:** Overview of automated tissue coverslippers, ordered by their maximum coverslipped slides per hour to indicate potential throughput. N/A denotes data undisclosed by the company.

Coverslipper Model	Vendor	Covering Material	Maximum Throughput [slides/hour]	Drying Time	Special Features	System Compatibility
Tissue-Tek Film Automated Coverslipper	Sakura Finetek Co. (Tokyo, Japan)	Film and xylene	1080	Approx. 4 s	No liquid mounting medium or bubbles, fast drying, histopathology and cytology specimens, air bubble detection	Tissue-Tek Prisma Plus Automated Slide Stainer
Tissue-Tek Glas g2 Automated Glass Coverslipper	Sakura Finetek Co. (Tokyo, Japan)	Glass	400	2 min	High reliability, precise dispensing of mounting medium to minimize air bubbles	Tissue-Tek Prisma Plus Automated Slide Stainer
HistoCore SPECTRA CV Coverslipper	Leica Biosystems (Nussloch, Germany)	Glass	570	5 min	Reduced bubble formation, high speed, for histology and cytology, integrated fume control	Leica HistoCore SPECTRA ST Stainer (integrated in HistoCore SPECTRA Workstation)
ClearVue Coverslipper	Epredia, a Thermo Fisher Scientific brand (Kalamazoo, Michigan, USA)	Glass	250	N/A	Intelligent control, smart automation, for histology and cytology, minimal bubbles	Gemini AS Automated Slide Stainer, Sakura DRS 2000, Leica Auto-Stainer
JQ-DS200 Slide Stainer and Coverslipper	Guangdong Jinquan Medical Technology Co. (Shenzhen, Guangdong, China)	Glass	200	N/A	Round-shaped tray, no xylene/alcohol, priority stain function, barcode reader	Staining and covering in one system

**Table 3:** Overview of different scanner types and commercially available products.

Category	Scanner Type/Application	Models
Whole-slide scanners	Brightfield	Leica Aperio GT450DX, 3DHISTECH Pannoramic 1000, Phillips Pathology Scanner SG300, Hamamatsu NanoZoomer S360
	Fluorescence	Visialab EPIQO
	Multimodal (brightfield + fluorescence)	Roche Ventana DP 600, ZEISS Axioscan, Akoya Biosciences PhenoImager HT, 3DHISTECH Flash
Advanced research imaging	Confocal microscopy	Nikon AX, Leica TCS SP8, 3DHISTECH Confocal
	Multiphoton microscopy	Olympus FVMPE-RS, ZEISS LSM 990 NLO
	Tissue Microarray (TMA)	3DHISTECH TMA Grand Master
Specialized imaging	Optical Coherence Tomography (OCT)	Thorlabs Vega Series SS-OCT systems, Heidelberg OCT Spectralis
	Portable/intraoperative	Grundium Ocus 40, OptraSCAN OS-FS Frozen Section Scanner
AI support decision software	Image analysis and decision support	Paige AI, PathAI, Aiforia, 3DHISTECH QuantCenter

**Table 4:** Overview of different scanner types and commercially available products, ordered by the scanning speed. Resolution and numerical aperture are specified for 40× magnification, and the scanning speed is reported for 40× magnification with a 15 mm × 15 mm Area of Interest (AOI).

Scanner Model	Vendor	Resolution [µm/pixel]	Numerical Aperture [NA]	Scanning Speed [seconds]	Slide Capacity	Use Case	FDA Approved
Pannoramic 1000	3DHISTECH Ltd. (Budapest, Hungary)	0.12	0.95	25	1000	Research	No
NanoZoomer S360	Hamamatsu Photonics K.K. (Hamamatsu, Japan)	0.23	0.75	30	360	Clinical	Yes
TissueScope iQ	Huron Digital Pathology (St. Jacobs, Ontario, Canada)	0.20	0.75	30	400	Research	No
Aperio GT450DX	Leica Biosystems (Nussloch, Germany)	0.26	0.75	32	450	Clinical	Yes
Pathology Scanner SG300	Philips Healthcare (Amsterdam, Netherlands)	0.25	0.75	43	300	Clinical	Yes
VENTANA DP 600	Roche Diagnostics GmbH (Mannheim, Germany)	0.23	n.a.	60	240	Clinical	Yes
Ocus40 (portable)	Grundium Oy (Tampere, Finland)	0.25	0.75	200	1	Research	No

**Table 5:** Summary of collaborative robotic arms in industrial and medical applications.

Robot Model	Vendor	Primary Application	DOF	Payload [kg]	Control Method	Sensors	Notable Features
UR5e	Universal Robots A/S (Odense, Denmark)	Collaborative assembly, pick-and-place	6	5	Impedance control, force–torque control	Integrated joint force–torque sensors	Intuitive graphical programming via teach pendant, built-in safety features, ideal for human–robot interaction.
UR20	Universal Robots A/S (Odense, Denmark)	High-payload tasks, palletizing	6	20	Impedance control, force–torque control	Integrated joint force–torque sensors	Long reach, suitable for heavier industrial tasks.
CRX-10iA	FANUC Corporation (Tokyo, Japan)	Material handling, welding	6	10	Collision-stop function, hand-guided teaching	Vision systems, torque sensors	Certified safety functions, can be powered by standard 120 V power.
GoFa CRB 15000	ABB Ltd. (Zürich, Switzerland)	High-payload tasks, machine tending	6	15	Lead-through programming, SafeMove software	Internal torque sensors	Built-in safety features, can work alongside humans without safety fences.
Franka Research 3	Franka Robotics GmbH (Munich, Germany)	Research, delicate tasks	7	3	Joint torque control, impedance control	Joint torque sensors, hand camera	Highly sensitive and lightweight, excellent for haptic applications, direct ROS integration, modular API.
LBR iiwa	KUKA Roboter GmbH (Augsburg, Germany)	Medical research, delicate assembly	7	14	Impedance control, direct teaching	Joint torque sensors	High-precision with integrated joint torque sensors, capable of delicate tasks, certified for collaborative operation.

**Table 6:** Mapping manual magazine manipulation tasks to movement types of robotic arm.

	Movement Types of Robotic Arm								
Interaction Steps with Stainer & Coverslipper	Trans. X	Trans. Y	Trans. Z	Rot. X	Rot. Y	Rot. Z	TCP Manipulator	Sum of DOF	Cumulative DOF
(a) Approaching to the coverslipper’s door	+	+	+	−	−	+	−	4	4
(b) Detecting the processed magazines through the door	−	−	−	−	−	−	LED, camera, CV, DIP	0	4
(c) Grabbing the closed door	+	+	+	−	−	+	Door gripper(+Trans.)	5	5
(d ↔ n) Opening/closing the door	+	+	+	+	−	−	Door gripper(+Trans.)	5	6
(e) Approaching to the processed magazines	+	+	+	+	−	+	−	5	6
(f) Identifying the earliest processed magazine	+	−	−	−	−	−	LED, camera, CV, DIP	1	6
(g, =ah) Grabbing a magazine	+	+	+	−	−	−	Magazine gripper(+Trans.)	4	7
(h, ↔ak) Removing a grabbed magazine from the stainer & coverslipper	+	+	+	+	−	−	Magazine gripper(+Trans.)	5	7
(i, =aj) Transporting a magazine (to the temporary holder)	+	+	+	−	−	−	−	3	7
(j, ↔ai) Inserting a magazine into the temporary holder	−	+	−	−	−	−	Magazine gripper(+Trans.)	2	7
(k, =al) Releasing a magazine	−	−	+	−	−	−	Magazine gripper(+Trans.)	2	7
(l) Returning to the opened door	+	+	+	+	−	+	−	5	7
(m) Grabbing the open door	+	+	+	−	−	−	Door gripper(+Trans.)	4	7
(o) Releasing the door	+	−	+	+	−	−	Door gripper(+Trans.)	4	7

Notes: The plus sign “+” denotes an active degree of freedom, while the minus sign “−” denotes an inactive degree of freedom of the robotic arm at a given workflow step. The symbol “↔” indicates that the corresponding motion step involves the same sequence of movements performed in opposite directions (e.g., opening vs. closing). The symbol “=” denotes motion steps that are kinematically equivalent but belong to different workflows.

**Table 7:** Mapping manual slide manipulation tasks to movement types of robotic arm.

	Movement Types of Robotic Arm								
Interaction Steps with Tissue Scanner	Trans. X	Trans. Y	Trans. Z	Rot. X	Rot. Y	Rot. Z	TCP	Sum of DOF	Cumulative DOF
(p, =ag) Approaching to a magazine in the temporary holder	+	+	+	−	−	+	−	4	7
(q) Detecting a transparent slide inside a magazine	−	−	−	−	−	−	LED, camera, CV, DIP	0	7
(r) Approaching to a detected slide	+	+	+	−	−	+	−	4	7
(s, ↔af) Grabbing a slide	+	−	+	−	−	−	Slide gripper (+Rot.,+Trans.)	4	9
(t, ↔ae) Removing a slide from a magazine	+	−	−	−	−	−	Slide gripper (+Rot.,+Trans.)	3	9
(u, =ad) Transporting a slide between the temporary holder and the tissue scanner	+	+	+	−	−	+	Slide gripper (+Rot.,+Trans.)	6	9
(v ↔ w) Inserting/removing a slide into/from the scanner layout rotator	+	+	+	+	−	−	Slide gripper (+Rot.,+Trans.)	6	9
(x, ↔ac) Transporting a slide to the robotic arm of the scanner	+	+	+	−	−	−	Slide gripper (+Rot.,+Trans.)	5	9
(y, ↔ab) Handling over a slide to the robotic arm of the scanner	−	−	+	−	−	−	Slide gripper (+Rot.,+Trans.)	3	9
(z, =aa) Moving the (empty) robotic arm in the scanner tunnel	+	+	+	−	−	−	−	3	9

Notes: The plus sign “+” denotes an active degree of freedom, while the minus sign “−” denotes an inactive degree of freedom of the robotic arm at a given workflow step. The symbol “↔” indicates that the corresponding motion step involves the same sequence of movements performed in opposite directions (e.g., opening vs. closing). The symbol “=” denotes motion steps that are kinematically equivalent but belong to different workflows.

**Table 8:** Mapping manual slide and magazine archiving tasks to movement types of robotic arm.

	Movement Types of Robotic Arm								
Archivation Steps	Trans. X	Trans. Y	Trans. Z	Rot. X	Rot. Y	Rot. Z	TCP	Sum of DOF	Cumulative DOF
(aa, =z) Moving the (empty) robotic arm in the scanner tunnel	+	+	+	−	−	−	−	3	9
(ab, ↔z) Taking a (scanned) slide from the robotic arm of the scanner	−	−	+	−	−	−	Slide gripper (+Rot.,+Trans.)	3	9
(ac, ↔x) Retracting the robotic arm with a (scanned) slide from the scanner tunnel	+	+	+	−	−	−	Slide gripper (+Rot.,+Trans.)	5	9
(ad, =u) Transporting a slide between the temporary holder and the tissue scanner	+	+	+	−	−	+	Slide gripper (+Rot.,+Trans.)	6	9
(ae, ↔t) Inserting a slide to a magazine	+	−	−	−	−	−	Slide gripper (+Rot.,+Trans.)	3	9
(af, ↔s) Releasing a slide	+	−	+	−	−	−	Slide gripper (+Rot.,+Trans.)	4	9
(ag, =p) Approaching a magazine in the temporary holder (from the front)	+	+	+	−	−	+	−	4	9
(ah, =g) Grabbing a magazine	+	+	+	−	−	−	Magazine gripper(+Trans.)	4	9
(ai, ↔j) Removing a magazine from the temporary holder	−	+	−	−	−	−	Magazine gripper(+Trans.)	2	9
(aj, =i) Transporting a magazine (to the local rack holder)	+	+	+	−	−	−	Magazine gripper(+Trans.)	4	9
(ak, ↔h) Placing a magazine onto the local rack holder	+	+	+	+	−	−	Magazine gripper(+Trans.)	5	9
(al, =k) Releasing a magazine	−	−	+	−	−	−	Magazine gripper(+Trans.)	2	9

Notes: The plus sign “+” denotes an active degree of freedom, while the minus sign “−” denotes an inactive degree of freedom of the robotic arm at a given workflow step. The symbol “↔” indicates that the corresponding motion step involves the same sequence of movements performed in opposite directions (e.g., opening vs. closing). The symbol “=” denotes identical motion, which can be found in more than one workflow.

**Table 9:** Comparison of average unit time requirements for each laboratory task in automatic and manual operation modes.

Average Unit Time Requirement	Automatic Mode [s]	Manual Mode [s]
(a) Approaching the door of the coverslipper	5	30
(b) Detecting the processed magazines through the door	1	3
(c) Grabbing the closed door	7	1
(d ↔ n) Opening/closing the door	11	5
(e) Approaching the processed magazines	8	1
(f) Identifying the earliest processed magazine	6	2
(g, =ah) Grabbing a magazine	8	2
(h, ↔ak) Removing a grabbed magazine from the stainer & coverslipper	8	4
(i, =aj) Transporting a magazine (to the temporary holder)	4	3
(j, ↔ai) Inserting a magazine into the temporary holder	12	14
(k, =al) Releasing a magazine	1	1
(l) Returning to the opened door	9	3
(m) Grabbing the open door	2	1
(o) Releasing the door	1	1
(p, =ag) Approaching a magazine in the temporary holder	7	2
(q) Detecting a transparent slide inside a magazine	3	3
(r) Approaching a detected slide	4	1
(s, ↔af) Grabbing a slide	5	2
(t, ↔ae) Removing a slide from a magazine	3	5
(u, =ad) Transporting a slide between the temporary holder and the tissue scanner	3	20–300
(v ↔ w) Inserting/removing a slide into/from the scanner layout rotator	8.5	-
(x, ↔ac) Transporting a slide to the robotic arm of the scanner	5	-
(y, ↔ab) Handling over a slide to the robotic arm of the scanner	3	-
(z, =aa) Moving the (empty) robotic arm in the scanner tunnel	4	-

**Table 10:** Key Performance Indicators of Robotic Arm.

Properties	Results
Trained laboratory task capacity	38 all/24 distinct
Minimum DOF necessary for the tasks	5 robot + 4 gripper = 9 DOF
Detected objects	Black magazines, transparent slides
Precision of object detection	1–2 pixel
Environment of detection, robustness	Real time, open-air, changes in ambient light
Dimension of object position determination	2D and 3D
Accuracy of position	$\bar{x}=0.16603$ mm, $\bar{y}=0.0935$ mm, $\bar{z}=0.47769$ mm
Interval of accurate position on image	Sphere around actual camera center, d = 20 mm
Total cycle time	260-slide batch staining approx. 40 min – 3 h (protocol-dependent) + coverslipping approx. 15 min + continuous, parallel magazine/slide transfer + slide scanning approx. 5–30 min/slide (resolution-dependent)
Human intervention during workflow	0
Approx. mean price [USD] (in 2025)	Robotic arm + custom gripper = 50.000

## Data Availability

The original contributions presented in this study are included in the article/Supplementary Materials. Further inquiries can be directed to the corresponding author.

## Supplementary Materials

The following supporting information can be downloaded from https://www.mdpi.com/article/10.3390/s25226830/s1, Figure S1: Rendered hardware design of the complete workflow created in Solid Edge (SolidEdge-Gemini_UR5_P1000_layout_design); Video S1: Demonstration of the early phase of magazine detection (Experimental_magazine_detection_phase_1 (.avi)); Video S2: Workflow feasibility proof that has already been patented (Gemini_automated_pathology_workflow (.mp4)).

## Abbreviations

The following abbreviations are used in this manuscript:

AI	Artificial intelligence
AOI	Area of interest
API	Application programming interface
CV	Computer vision
cobot	Collaborative robot
DIP	Digital image processing
DOFs	Degrees of freedom (robotic arm)
GUI	Graphical User Interface
H&E	Hematoxylin and Eosin
IHC	Immunohistochemistry
OCT	Optical Coherence Tomography
ROS	Robot Operating System
Rot X/Y/Z	Rotation movement with robotic arm in X/Y/Z axis
TCP	Tool Central Point
TDI	Time Delay Integration
TMA	Tissue microarray
Trans X/Y/Z	Translation movement with robotic arm in X/Y/Z axis
WSI	Whole slide imaging
