# Supplementary material for: Mapping Manual Laboratory Tasks to Robot Movements in Digital Pathology Workflow
Source: Sensors (Basel). 2025 Nov 8;25(22):6830. doi: 10.3390/s25226830 (PMC12655976; doi:10.3390/s25226830)
Supplement: Supplementary file 1 [file sensors-25-06830-s001.zip › SI-sensors-3938809.pdf]

sensors

Article

Mapping Manual Laboratory Tasks to Robot Movements in Digital Pathology Workflow

Marianna Dimitrova Kucarov [1,2,3,*], Mátyás Takács [3,4], Bence Géza Czakó [5], Béla Molnár [3,6] and Miklos Kozlovszky [2,7,8]

1 Doctoral School of Applied Informatics and Applied Mathematics, Obuda University, 1034 Budapest, Hungary
2 BioTech Research Center, Obuda University, 1034 Budapest, Hungary; kozlovszky.miklos@uni-obuda.hu
3 3DHistech Ltd., 1141 Budapest, Hungary; matyas.takacs@3dhistech.com (M.T.); molnar.bela@semmelweis.hu (B.M.)
4 Antal Bejczy Center for Intelligent Robotics (IROB), Obuda University, 1034 Budapest, Hungary
5 Turbine Ltd., 1083 Budapest, Hungary; bence.czako@turbine.ai
6 Department of Internal Medicine and Oncology, Faculty of Medicine, Semmelweis University, 1085 Budapest, Hungary
7 John von Neumann Faculty of Informatics, Obuda University, 1034 Budapest, Hungary
8 Medical Device Research Group, LPDS, Institute for Computer Science and Control (SZTAKI), Hungarian Research Network (HUN-REN), 1111 Budapest, Hungary
* Correspondence: kucarov.marianna@uni-obuda.hu

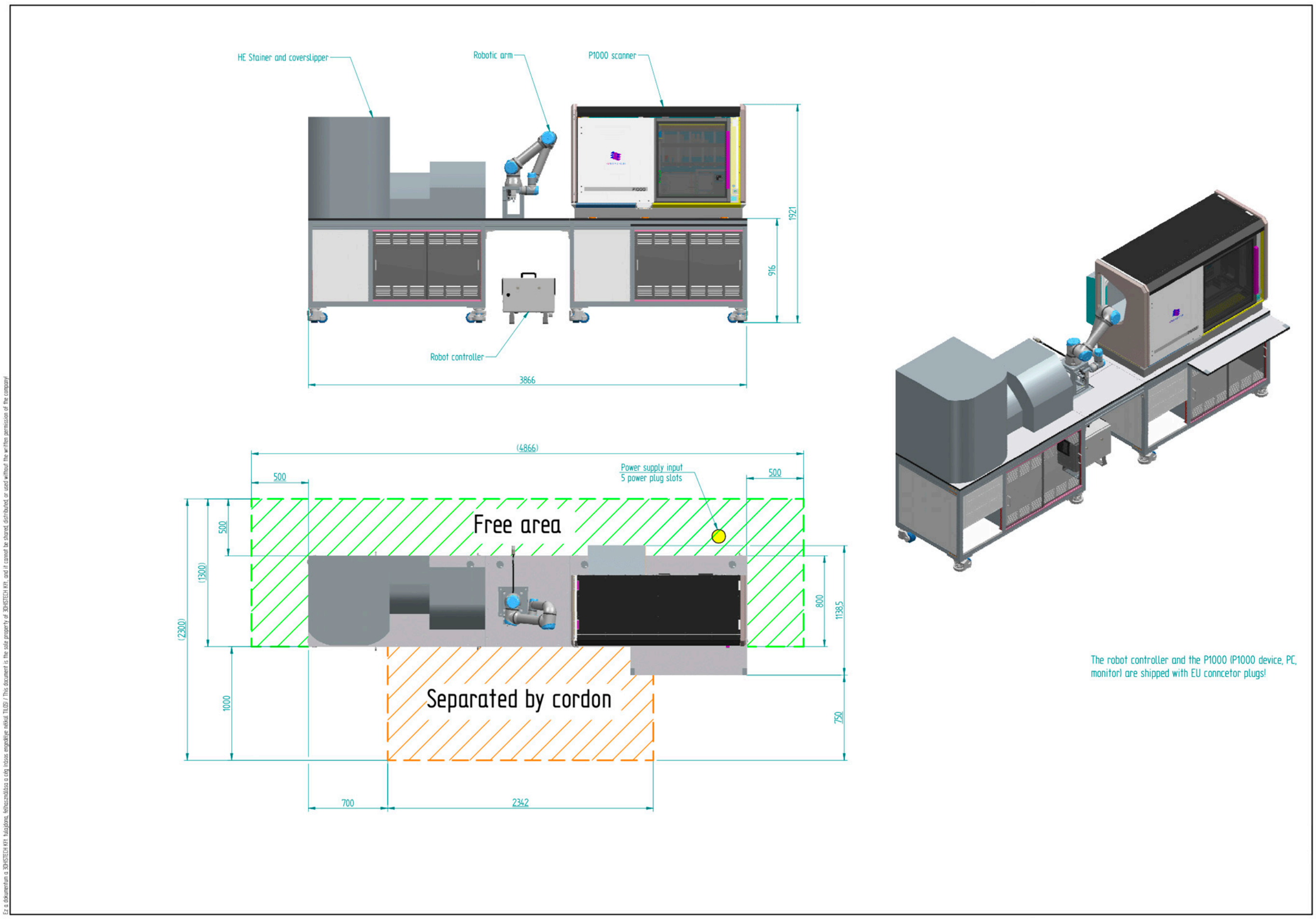

Figure S1. Gemini-UR5-P1000 layout design created in Solid Edge.

 https://doi.org/10.3390/s25226830
